# Bioproduction of 2-Phenylethanol through Yeast Fermentation on Synthetic Media and on Agro-Industrial Waste and By-Products: A Review

**DOI:** 10.3390/foods11010109

**Published:** 2022-01-01

**Authors:** Sara Mitri, Mohamed Koubaa, Richard G. Maroun, Tristan Rossignol, Jean-Marc Nicaud, Nicolas Louka

**Affiliations:** 1 Université de Technologie de Compiègne, ESCOM, TIMR (Integrated Transformations of Renewable Matter), Centre de Recherche Royallieu, CS 60319, CEDEX, 60203 Compiègne, France; sara.mitri@net.usj.edu.lb; 2 Laboratoire CTA, UR TVA, Centre d’Analyses et de Recherche, Faculté des Sciences, Université Saint-Joseph, Beyrouth 1104 2020, Lebanon; richard.maroun@usj.edu.lb (R.G.M.); nicolas.louka@usj.edu.lb (N.L.); 3 Université Paris-Saclay, INRAE, AgroParisTech, Micalis Institute, 78350 Jouy-en-Josas, France; tristan.rossignol@inrae.fr (T.R.); jean-marc.nicaud@inrae.fr (J.-M.N.)

**Keywords:** 2-phenylethanol, agro-industrial waste, biotechnology, microbial fermentation, yeasts, natural aromas, in situ product recovery

## Abstract

Due to its pleasant rosy scent, the aromatic alcohol 2-phenylethanol (2-PE) has a huge market demand. Since this valuable compound is used in food, cosmetics and pharmaceuticals, consumers and safety regulations tend to prefer natural methods for its production rather than the synthetic ones. Natural 2-PE can be either produced through the extraction of essential oils from various flowers, including roses, hyacinths and jasmine, or through biotechnological routes. In fact, the rarity of natural 2-PE in flowers has led to the inability to satisfy the large market demand and to a high selling price. Hence, there is a need to develop a more efficient, economic, and environmentally friendly biotechnological approach as an alternative to the conventional industrial one. The most promising method is through microbial fermentation, particularly using yeasts. Numerous yeasts have the ability to produce 2-PE using l-Phe as precursor. Some agro-industrial waste and by-products have the particularity of a high nutritional value, making them suitable media for microbial growth, including the production of 2-PE through yeast fermentation. This review summarizes the biotechnological production of 2-PE through the fermentation of different yeasts on synthetic media and on various agro-industrial waste and by-products.

## 1. Introduction

Over the years, fragrance usage became ubiquitous. A huge number of flavors and fragrances found their way into the daily life of almost all human beings. 2-Phenylethanol (2-PE), also known as 2-phenethyl alcohol, is a higher aromatic alcohol characterized by one of the most popular and desired fragrances, which is the rosy scent [1]. 2-PE has a wide range of applications in diverse types of products. It is commonly used in cosmetics including perfumes, in pharmaceuticals as a preservative and in various herbal products. Additionally, 2-PE is used in food and beverage industries as organoleptic enhancer of the final product [2,3]. For instance, this aroma is added to the composition of a variety of ice creams, candies, cookies, puddings, gelatins, cigarettes, and chewing gums [4]. This flavor has been approved by many worldwide organizations, including the Food and Drug Administration (FDA), Flavor and Extract Manufacturers’ Association (FEMA), the Joint Expert Committee on Food Additives (JECFA), and the Council of Europe (COE). They consider 2-PE as generally recognized as safe (GRAS, 2858), which gives it an added value [5]. Additionally, 2-PE exerts antimicrobial and antifungal properties. It can inhibit the growth of several Gram-positive and Gram-negative microorganisms, such as *Escherichia coli*, *Staphylococcus aureus*, *Enterococcus faecium* and many fungal species including *Candida albicans*, *Candida dubliniensis*, *Saccharomyces cerevisiae*, *Kluyveromyces marxianus*, and many other microorganisms [6]. These characteristics made this substance an appreciated additive in antiseptics, preservatives, cleaning, and personal care products [2]. It is noteworthy that 2-PE is also being used in aromatherapy. It was demonstrated that the odor of rose oil reduces plasma adrenaline concentration by 30% and human sympathetic activity by 40% [7]. In addition, 2-PE is used as a precursor for the synthesis of other valuable chemicals such as 2-phenethyl acetate (2-PEA), which is a volatile ester having also a rose-like odor [8].

The world production of 2-PE is approximately 10,000 tons per year, most of which is obtained by chemical synthesis, with a price between 3.5 and 5 US$/kg [9,10]. However, natural 2-PE, extracted from the essential oil of some flowers, is much more expensive compared to the chemically synthesized 2-PE with an estimated price of about 1000 US$/kg [11]. Martinez-Avila et al. estimated that the bioproduction of 2-PE via a microbial route would actually cost around 220 US$/kg [12].

Three different paths are used in the industry to chemically synthesize 2-PE. First, the Friedel-Crafts reaction of ethylene oxide with benzene in the presence of aluminum chloride. Second, the hydrogenation of styrene oxide with a small amount of sodium hydroxide and Raney nickel as a catalyst. Third, the oxidation of propylene with 2-phenylethyl hydroperoxide [13]. These methods involve the use of toxic chemicals (e.g., benzene, styrene oxide), which are carcinogenic and hazardous to the health and environment. Furthermore, chemical synthesis needs high temperatures, high pressure, and strong alkali or acid conditions. It is also associated with the production of unwanted byproducts. All of this, affects the quality of the final product, making its purification difficult [14,15].

On the other hand, natural 2-PE can be extracted from the essential oils of various flowers, including jasmine, hyacinth, lilies, and daffodils. However, the concentration of 2-PE in these flowers is too low, except for rose oil which can contain up to 60% 2-PE [11]. For instance, the dominant aroma compound produced by *Rosa damascena*, also known as damask roses, is 2-PE [16]. Because of the low concentration of 2-PE in almost all types, multiple separation steps are required [12]. As a consequence of the aforementioned rarity of natural 2-PE in flowers, requiring complicated and costly downstream processing, the large market demand cannot be satisfied [14].

The uses of 2-PE in cosmetic products and in different foods and pharmaceuticals drove an increased demand for natural methods of production of this aroma [17], thus boosting research to find alternative methods for the production of natural 2-PE through biotechnological approaches [18]. The most promising method is through microbial fermentation, mainly the one based on the synthesis of 2-PE using yeasts [19]. Yeasts have the ability to synthesize 2-PE using l-phenylalanine (l-Phe) or glucose as substrates using the Ehrlich pathway and the shikimate pathway, respectively [4,20]. In fact, biotechnological production of flavors and fragrances is becoming more attractive, since it produces a final product classified as natural by the US Food and Drug Administration and by the European legislation, if the substrates used in the process are of natural origin [21]. In addition to the formation of a “natural” product, the production cycle of microbial fermentation is short and environmentally friendly, decreasing the environmental pollution caused by the chemical synthesis of 2-PE [22,23]. However, it is noteworthy that microbial fermentation techniques need highly productive yeasts and cheap feedstocks in order to be economically attractive and compete with the chemical synthesis routes [24]. Agro-industrial waste and by-products having high nutritional value offer suitable environments for the growth of microorganisms. Through fermentation, microorganisms have the ability to use them as potential raw materials for the production of value-added products [25]. This is known as “biorefinery”, whereby the waste and by-products of an industry can serve as the raw material for another [26].

In this review, the biotechnological production of 2-PE through the fermentation of different yeasts on various agro-industrial waste and by-products as feedstocks will be discussed. A description of the yeasts capable of producing 2-PE, the metabolic pathways responsible for 2-PE biosynthesis, and the production of 2-PE on synthetic media will be highlighted. Finally, the utilization of agro-industrial waste and by-products as feedstocks for 2-PE production, as well as the strategies used to increase 2-PE productivity will be discussed.

## 2. Yeasts Producing 2-PE

Yeasts are microorganisms with large synthetic ability. Through enzyme-catalyzed reactions, they can convert simple carbohydrates and nitrogen sources into different complex molecules, particularly various flavor compounds [27]. In fact, numerous yeast species have the ability to produce 2-PE [9], and this production is strain specific, which means that a significant difference in 2-PE production level may be seen between different strains of one species [28]. It is noteworthy that recently numerous metabolically engineered yeast strains have been constructed in order to significantly enhance 2-PE yield [29].

*Saccharomyces cerevisiae*, a significant eukaryotic model organism, is a well-known promising microorganism for its ability to produce 2-PE [30]. Many studies have proved that other so-called non-conventional yeasts are also able to produce 2-PE and some of them show a higher capacity for aroma metabolite production compared to *S. cerevisiae* [31]. *Kluyveromyces marxianus* [19], *Kluyveromyces lactis*, *Pichia fermentans*, *Pichia anomala*, *Pichia membranaefaciens*, *Candida utilis* [32], *Phellinus ignarius*, *Ischnoderma benzoinum*, *Geotrichum penicillatum*, *Aspergillus niger* [33], *Meyerozyma caribbica*, *Meyerozyma guilliermondii*, *Metschnikowia chrysoperlae*, *Clavispora lusitaniae*, *Candida tropicalis* [24], *Yarrowia lipolytica*, *Zygosaccharomyces rouxii*, *Pichia kudriavzevii* [2], and *Pichia pastoris* have all been previously reported to produce 2-PE, but to different extents [34].

Later in this review, the ability of conventional and non-conventional yeasts to produce 2-PE on synthetic media and on agro-industrial waste and by-products will be discussed, along with the factors and fermentation conditions affecting the concentration of 2-PE produced by each species.

## 3. Biochemical Pathways for 2-PE Production

**Figure 1:**
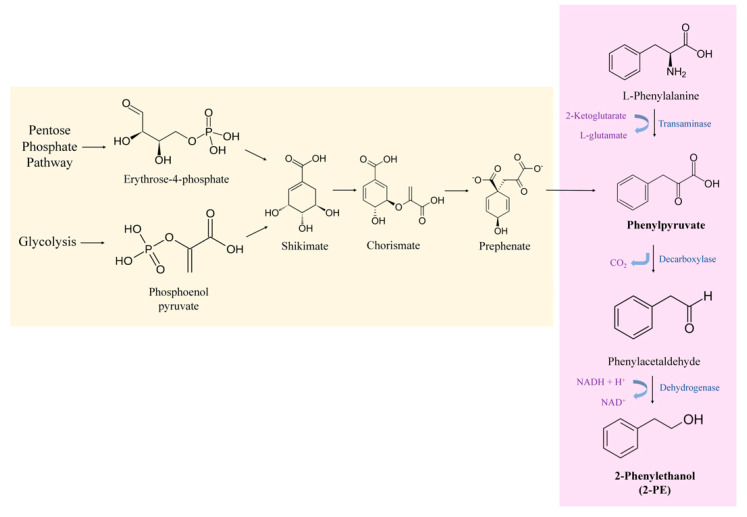
Metabolic pathways involved in the production of 2-PE in yeasts: Shikimate pathway for de novo synthesis of 2-PE and Ehrlich pathway for 2-PE production from l-Phe.

Yeast cells are capable of producing 2-PE via normal metabolism. 2-PE can be synthesized through two independent routes in yeast cells, either de novo via the shikimate pathway or by the bioconversion of l-Phe via the Ehrlich pathway (Figure 1) [35].

The Ehrlich pathway is considered as the most efficient biotechnological approach and thus the more industrially attractive option. Most advances in biotechnology have focused on the development of this process [18]. In this mechanism, the yeast cells use the aromatic amino acid, l-phenylalanine, as sole nitrogen source in order to produce 2-PE. It involves three steps, where the first one consists of converting l-Phe into phenylpyruvate. In the model yeast *S. cerevisiae*, this transamination reaction is catalyzed by two amino acid transaminase isoenzymes Aro8p and Aro9p. Second, phenylpyruvate is decarboxylated to phenylacetaldehyde. The second step of the Ehrlich pathway is catalyzed by the phenylpyruvate decarboxylase enzyme, Aro10p, and the three pyruvate decarboxylase isoenzymes Pdc1p, Pdc5p and Pdc6p. Finally, the third step consists of reducing the phenylacetaldehyde to 2-PE by alcohol dehydrogenases (Adh1p, Adh2p, Adh3p, Adh4p, Adh5p) and formaldehyde dehydrogenase Sfa1p [30,35]. This natural process is significantly improved when the amino acid l-Phe is available in the media, since high concentrations of l-Phe are needed to shift cell metabolism to the Ehrlich pathway [36].

On the other hand, yeasts can produce 2-PE from intermediate molecules of their metabolism (e.g., phosphoenolpyruvate (PEP) and erythrose-4-phosphate (E4P)). This is known by the de novo synthesis of 2-PE which takes place through the shikimate pathway. In this pathway, simple sugars are transformed into 2-PE [37]. PEP and E4P resulting from glycolysis and pentose phosphate pathway, respectively, are catalyzed to synthesize 3-deoxy-D-arabinoheptulosonate-7-phosphate (DAHP). Then, through a series of intermediates including shikimate, chorismate, prephenate, etc., phenylpyruvate is produced (Figure 1). Phenylpyruvate, the product of the shikimate pathway, next enters in the Ehrlich pathway. Hence, phenylpyruvate is then decarboxylated to phenyacetaldehyde and dehydrogenated to 2-PE [38]. It is noteworthy that shikimate pathway involves many steps with branched metabolic pathways and a variety of feedback inhibitions. Moreover, the glycolysis and the pentose phosphate pathways are mainly utilized for cell growth rather than 2-PE production. This will limit the transformation of simple sugars to 2-PE, hence the final concentration of 2-PE obtained is very low [35].

## 4. Factors Affecting the Rate of 2-PE Production in Yeasts

The extent to which yeasts are capable of producing 2-PE does not only depend on the species itself, but also on the media composition and the fermentation conditions [4].

If amino acids, particularly l-Phe, are the sole nitrogen source in the medium, the Ehrlich pathway predominates over de novo synthesis, which usually dominates at low amino acid concentrations [39]. However, in the presence of more assimilable nitrogen sources, l-Phe will be metabolized through the cinnamate pathway, limiting the 2-PE formation. Therefore, high concentrations of 2-PE can be achieved by supplying l-Phe as a sole nitrogen source [40]. It is important to note that the usage of l-Phe as a substrate for 2-PE production is costly [12]. Therefore, metabolic engineering is being used to boost the aromatic amino acids pathway (AAA) for providing l-Phe for the bioconversion and also overexpression of key enzymes of the Ehrlich pathway [41].

On the other hand, fermentation conditions, particularly the availability of nutrients, nitrogen sources, and sugars supplied in the culture media, affect the metabolic activity of the microorganisms [42]. Additionally, temperature, pH, oxygen availability, and air flow-rate greatly affect 2-PE production by altering the microbial growth, the microbial community structures, or the key enzyme activities [15].

## 5. 2-PE Production via Fermentation of Yeasts on Synthetic Media

Various yeast species were capable of producing different amounts of 2-PE during growth on synthetic media. A study done by Chreptowicz et al. showed that *S. cerevisiae* JM2014 strain, a non-genetically modified strain that was isolated from a fermented milk drink, was able to produce 3.6 g/L of 2-PE after 72 h incubation at 30 °C. The batch culture was done in a 6.2 L bioreactor containing 4 L of medium 8 and 5 g/L l-Phe. Medium 8 is specific for l-Phe biotransformation to 2-PE and its major constituents are 15 g/L glucose, 8 g/L sucrose, and 5 g/L l-Phe [43]. Another study done by de Lima et al. reported that the wild-type (WT) *K. marxianus* CCT 7735 strain was able to produce 3.44 g/L of 2-PE under optimized conditions. The batch culture was done in 125 mL Erlenmeyer flask containing 25 mL synthetic medium (3 g/L glucose and 4 g/L l-Phe) at 30 °C, with a stirring rate of 200 rpm for 72 h [44]. Additionally, the WT strain *C. glycerinogenes* WL2002-5 was able to produce 5 g/L of 2-PE under optimized conditions. Batch fermentation was done in a 5 L bioreactor containing 3 L of synthetic medium supplemented with 90 g/L glucose and 7 g/L l-Phe for 50 h at 30 °C and 500 rpm [45]. Furthermore, the WT *Clavispora lusitaniae* WUT17 was able to produce 2.04 g/L of 2-PE in medium 8. The experiment was done in batch cultures incubated at 30 °C and 240 rpm for 72 h [46]. WT *Pichia kudriavzevii* YF1702 was reported to produce 5.09 g/L of 2-PE under its optimal fermentation conditions. Fermentation was done in 250 mL flasks containing 25.5 mL cultivation media (50 g/L glucose and 10.7 g/L l-Phe) for 56 h at 26 °C and 210 rpm shaking [47]. *Z. rouxii* M2013310 strain was able to produce 3.58 g/L 2-PE after 72 h fermentation in M3 culture medium containing 30 g/L glucose, 8 g/L sucrose and 9 g/L l-Phe [48].

Additionally, a *S. cerevisiae* CWY132 mutant strain was able to produce 3.76 g/L 2-PE under optimized conditions. Fermentation was done in a batch process with medium containing 34.16 g/L glucose and 5 g/L l-Phe [49]. Another study reported that *S. cerevisiae* PFP-26, a mutant strain resistant to the phenylalanine analogue p-fluoro-d,l-phenylalanine (PFP) and isolated from the parent yeast *S. cerevisiae* Kyokai No.9 was able to produce 0.973 g/L. *S. cerevisiae* PFP-26 cells were shaken in MM5 medium at 30 °C for 48 h. MM5 is a minimal medium containing 5% glucose [50].

On the other hand, several genetic engineering strategies were developed in order to improve 2-PE production in various yeast strains. A study done by Kim et al. reported that a genetically engineered *S. cerevisiae* strain, JHY315 strain, was able to produce 4.8 g/L of 2-PE. JHY315 is an aldehyde dehygrogenase 3 (*ALD*3) deletion strain overexpressing *ARO9* and *ARO10* genes by episomal overexpression and overexpressing the transcription factor Aro80 in order to induce the endogenous genes. This strain was grown on a synthetic media composed mainly of 20 g/L glucose and 10 g/L l-Phe amino acid. The batch culture was done in 250 mL shake flask and cultivated at 30 °C with shaking at 250 rpm [51]. Another study done by Kim et al. showed that the genetically modified *K. marxianus* BY25569 strain, overexpressing a feedback resistant (fbr) DAHP synthase, known as AroG^fbr^, from *Klebsiella pneumoniae* and hence improving the production of DAHP from glucose, can produce 1.3 g/L of 2-PE without the presence of l-Phe. The inoculum was cultivated in 5 mL of defined synthetic medium, containing 20 g/L glucose [52]. Additionally, a novel promising 2-PE producer is *Y. lipolytica*. At 95 h after the addition of the l-Phe, the genetically engineered *Y. lipolytica* NCYC3825 strain produced 1.98 g/L of 2-PE. Bioconversion of l-Phe to 2-PE was done in shake flasks containing the cultivation media, containing mainly 40 g glucose, and supplemented with 7 g/L l-Phe after 73 h of cultivation [9,53]. Finally, a *P. pastoris* SK004 strain overexpressing *ARO10*, *ARO8*, the aldehyde reductase *ADH6* gene, *aroG^fbr^* and chorismate mutase-prephenate dehydratase (*pheA^fbr^*) which is a feedback resistance mutant gene. *aroG^fbr^* and *pheA^fbr^* were previously reported, in the bacterium *E. coli*, to reduce the feedback inhibition of l-Phe biosynthesis by the accumulated phenylpyruvate. After 36 h incubation at 30 °C, in a rotary shaker at 200 rpm, this strain was able to produce 1.169 g/L 2-PE [34].

Subsequently, various yeasts were able to produce 2-PE to different extents, depending on the characteristics and type of the species and strain used and the nutrients present in the synthetic media. Based on the studies mentioned above, it can be noted that the different yeasts growing on multiple synthetic media were able to produce between 1.17 and 5.08 g/L 2-PE. An important factor being the amount of l-Phe. Increasing the supply of l-Phe leads to a higher production of 2-PE. It is noteworthy that the key parameter of 2-PE production is to have strains capable of producing 2-PE through their normal metabolism, however, l-Phe supplementation to the medium allowed higher production of 2-PE [40].

Additionally, conventional culture media are expensive due to their constituents particularly some amino acids and the gelling agents, such as l-Phe and agar, respectively. Therefore, these media are not cost-effective at a large-scale, and industries have started to search for and use various alternative cheap carbon sources (Table 1) [54].

## 6. 2-PE Production via Fermentation on Agro-Industrial Waste and By-Products

Agri-food industries produce a huge quantity of residues every year. In fact, one third of the food produced worldwide for the human consumption is lost or wasted annually, which is approximately 1.3 billion tons [26]. Since food waste is reaching alarming levels nowadays, ways of reusing it have been continuously developed and improved. Actually, most of the agro-industrial waste and by-products have high nutritional composition such as complex carbohydrates, proteins, lipids, and many nutraceuticals [55,56]. Therefore, these residues represent a high potential to be valorized. The availability of these nutrients in these raw materials exhibit proper environments for the growth of different microorganisms, particularly yeasts. Through fermentation, microorganisms have got the ability to utilize these raw materials in order to produce high-value products [25]. The utilization of agricultural and food by-products as cheaper substrate for the production of 2-PE have various advantages including the abundant availability of the substrates and their richness in nutrients, the reduced cost of production of this aroma molecule, the ability to produce a “natural” product, and the environmentally friendly process (Table 1) [19].

The most common bioprocess used to transform l-Phe into 2-PE is through submerged fermentation (SmF). SmF uses sterilized synthetic media as substrates and consists of complex reaction systems that consumes big amounts of key resources to attain high titers, hence considered an energy consuming and costly productive process [12]. An alternative technology that is gaining attention in the past few years is known as solid-state fermentation (SSF). SSF is usually used to produce high-value added molecules by fungi, but it has been also proposed to obtain 2-PE sustainably and economically from renewable sources [57]. SSF commonly present high production yields and rates with quite low energy and water requirements. It is energy efficient and produces limited hazardous materials and waste [12]. Many factors affect the SSF including moisture and water activity, the aeration strategy, pH and temperature of the media, the carbon and nitrogen sources, and the particle size of the substrate. Finally, SSF has proved its efficiency as an economical and environmentally friendly tool for the valorization of various organic solid waste in order to produce valuable aroma compounds [58]. Generally, SSF systems are batch processes in which the substrates, enzymes, and yeasts are all loaded in the reactor entirely at the beginning of the process. This approach is effective at lab scale, but at higher scales, alternative operational strategies, such as fed-batch mode, have been evaluated to enhance SSF performances. In fed-batch mode the substrate and l-Phe are not loaded completely at the beginning, instead they are added systematically. Particularly, the load is split into three parts at different time points throughout the fermentation process. Operating in fed-batch is also a tool to enrich 2-PE production in liquid cultures. Around 50% increase in 2-PE production levels was achieved in fed-batch compared to batch mode [12,59]. Many studies have been carried out to test the ability of different yeast species to ferment various agro-industrial waste and by-products and use them as raw materials to produce 2-PE.

### 6.1. Whey

Around 160 million tons of whey are annually produced, mainly by Europe and the United States [23]. It is a by-product of the cheese production industries. Whey has a low cost ranging between 0.40 US$/kg and 0.9 US$/kg [60]. It has around 55% of the total milk ingredients including lactose, soluble proteins, lipids, and mineral salts. Eventually, the high lactose content in whey makes it a potential raw material for the production of 2-PE [13]. Not all yeast species are capable of fermenting lactose. In fact, it was reported that *K. marxianus* has the ability to use lactose as carbon source and produce high concentrations of 2-PE [24]. 

A study done by Conde-Báez et al. showed that *K. marxianus* ITD00262 strain was able to produce 0.96, 0.7, and 0.47 g/L of 2-PE after 24 h in sweet whey, acid whey, and curd whey, respectively, in the absence of l-Phe [23]. Another study done by Chreptowicz et al. showed that *Barnettozyma californica* WUT11, *Candida lusitaniae* WUT17, *Metschnikowia chrysoperlae* WUT25, *Metschnikowia* sp. WUT12, WUT14, WUT26, *Meyerozyma guilliermondii* WUT22, *Meyerozyma caribbica* WUT28, *Pichia kluyveri* WUT2, *Pichia fermentans* WUT36, *Pichia kudriavzevii* WUT7, and *Wickerhamomyces anomalus* WUT9 are capable of producing between 1 and 2 g/L of 2-PE when fermented for 72 h batch cultures in shaking glass tubes containing WGP medium (3% whey, 2% glucose, 0.5% l-Phe). Under the same conditions, *S. cerevisiae* strains JM2014, WUT4, WUT34, and WUT35 were able to produce more than 2 g/L of 2-PE. Interestingly, in the same study some species were able to produce more 2-PE in WGP medium compared to synthetic medium 8, namely *Hanseniaspora opuntiae* WUT19, *Met. chrysoperlae* WUT25, *P. fermentans* WUT36, *Rhodotorula mucilaginosa*, *S. cerevisiae* WUT34 and WUT35. All the strains used in this study were not genetically modified [24].

### 6.2. Grape Must

Grape must, which is the freshly pressed grape juice, is a complex chemical matrix. This medium depends on the grape vineyard, grape types, ripeness stage, climate, terroir characteristics, and viticultural factors [61]. Water is the most abundant substance in grape must, followed by sugars (mainly glucose and fructose). It also contains organic acids, mineral salts, nitrogen substances, phenolic compounds, proteins, and vitamins [62]. In Europe alone, a total of 14.5 million tons of grape by-products, including grape musts, are produced annually [63]. In fact, the price of bulk grape must, in Spain in 2019, was around 0.3037 €/L [64].

In a study done by Garavaglia et al., the *K. marxianus* CBS 6556 strain was grown in grape must medium. It was able to produce 0.39 g/L of 2-PE after 84 h cultivation at 30 °C and pH 6.5 in shake flasks. Subsequently, the conditions of this experiment were optimized. *K. marxianus* CBC 6556 was cultivated again in grape must medium at pH 7, 37 °C temperature, and an initial l-Phe concentration of 3.0 g/L. The agitation speed was set at 250 rpm and the air flow rate was set to 1 vvm. After optimizing the experimental conditions, *K. marximus* CBC 6556 was able to produce 0.77 g/L of 2-PE. It was previously reported that the same strain was able to produce 0.21 g/L of 2-PE in a synthetic medium supplied with yeast nitrogen base and 3 g/L of l-Phe [4]. Additionally, Etschmann et al. mentioned that *Torulopsis utilis* and *Saccharomyces vini* were able to produce 12 mg/L of 2-PE each in 7 days fermentation on grape must [40].

### 6.3. Corn Stover

Corn stover is one of the main crop straws. Particularly, it represents 35% of the global annual production of straw which is estimated to be 2.9 billion tons. Corn stover is generally a cheap abundant material, widely distributed, and multi-sourced. It costs around 0.083 US$/kg of corn stover. It is not profusely used and hence presents a great potential for development and utilization [65,66]. This agro-industrial residue, contains high levels of lignocellulose and is considered to be a promising low-cost natural energy source for the bioproduction of 2-PE and a promising feedstock for bioethanol production [67].

In order to be able to utilize this by-product as a substrate for microbial fermentation and production of 2-PE, the yeasts used should have the capacity to metabolize the monosaccharide xylose, which is abundant in corn stover. *P. fermentans* WUT36 and *Met. chrysoperlae* WUT25 are able to metabolize xylose and convert the l-Phe into 2-PE. At first, corn stover biomass needs to be pretreated and hydrolyzed in order to release the fermentable monosaccharides. Then, *P. fermentans* WUT36 and *Met. chrysoperlae* WUT25 are fermented in a batch culture for 72 h in a medium containing lignocellulosic hydrolysate, yeast extract, and l-Phe. *P. fermentans* WUT36 produced between 2.23 and 2.97 g/L of 2-PE while *Met. chrysoperlae* WUT25 produced between 1 and 1.11 g/L of 2-PE, depending on the pretreatment time of the lignocellulosic hydrolysate. Next, enlargement of the culture volume from 25 mL to 250 mL of medium, at a 1000 mL flask scale, was tested in order to further examine the relevancy of the corn stover hydrolyzates as a feedstock in 2-PE production. The highest 2-PE concentration was produced by *P. fermentans* WUT36 and was equal to 3.67 g/L of 2-PE [19].

### 6.4. Sugar Beet Molasses (SBM)

Molasses is a cheap and abundant by-product of sugar industry. It is a viscous dark liquid that contains large amounts of sugars; mainly around 47 to 48%, in which sucrose is the major fermentable sugar. The European Union produces 17 million tons of beet sugar annually, from which 6 million tons of molasses are produced. The estimated price of beet molasses is around 0.132 US$/kg. Molasses have been used for microbial production of 2-PE [68,69,70,71].

A study done by Etschmann et al. tested for the ability of multiple yeast species to use molasses as a carbon source to produce 2-PE. Fourteen different strains of yeasts were tested including *K. marxianus*, *K. lactis*, *Z. rouxii*, *S. cerevisiae*, *C. lusitaniae*, *P. anomala*, *P. membranaefaciens*, and *S. pombe*. Eleven out of the fourteen strains were able to produce between 0.13 and 0.89 g/L of 2-PE using sugar beet molasses at 35 °C. The most productive strain was *K. marxianus* CBS 600 with a yield of 0.89 g/L after 41 h [39]. Other studies have shown that *S. cerevisiae* can produce up to 4 g/L of 2-PE using SBM. Moreover, *P. fermentans* can produce 1.88 g/L of 2-PE in mixed media of cheese whey and sugar molasses [2].

### 6.5. Sugarcane Bagasse (SCB)

Sugarcane bagasse is another by-product of the sugar industry. Particularly, it is the residual substance of sugarcane stems that remains after juice extraction, which is known as sugarcane milling. Out of each ton of processed sugarcane, around 270 to 280 kg of bagasse is produced. SCB is mainly composed of cellulose, glucan, hemicellulose, lignin, extractives, and ashes [70]. According the United Nations data, 493 million metric tons of bagasse are produced annually from the sugar industry [72]. Sugarcane bagasse price is highly variable ranging between 8.11 to 40.5 US$/t [73]. This abundant agro-industrial waste can be used as substrate for fermentation processes [74].

A study done by Martínez-Avila et al. tested the ability of a *Pichia kudriavzevii* strain with interesting characteristics that favor 2-PE bioproduction via SSF. In addition to its natural ability to produce 2-PE via SSF, this *P. kudriavzevii* strain exhibited low-pH tolerance, high growth rate, and temperature resistance which allowed it to process liquid and solid waste streams and use them as substrate. Therefore, by using this strain there was no need for balancing the medium pH, adding micronutrients to it, or using high inoculum load in order to obtain high 2-PE concentrations and productivities. Under optimized conditions, *P. kudriavzevii* CECT 10467 was able to produce 27.2 mg of 2-PE per gram of dry substrate. Batch tests were conducted at 31 °C, 76% initial moisture content and 0.129 L/h/g specific air flow rate [58]. Other studies have proven that *K. marxianus* is also able to produce 2-PE using the SCB [74].

### 6.6. Tobbaco

In 1995, the global tobacco manufacturing industries produced around 2 million tons of solid waste which have detrimental effects on the environment [75]. Nowadays, new opportunities for utilization of tobacco and valorization of tobacco waste are emerging. In fact, they are being used as a potential feedstock to synthesize bio-based products, including 2-PE. A study done by Wang et al. showed that *S. cerevisiae* was able to produce a maximum amount of 1.55 g/L of 2-PE on 39.28 g/L of tobacco waste and at pH 5.9 [33].

### 6.7. Cassava Wastewater (CWW)

Cassava wastewater is an agro-industrial waste produced by the starch industry. It is generated in large volumes and contains high levels of organic compounds and cyanogenic glycosides. The annual production of cassava starch in Colombia is 12 thousand tons, which generates between 60 and 90 m^3^ of wastewater per ton of starch [76]. CWW was studied for the possibility of utilizing it as a substrate for 2-PE production. Oliveira et al. demonstrated that a *S. cerevisiae* strain cultivated in an optimized batch culture of 50 mL medium, supplemented with 20 g/L glucose and 5.5 g/L l-Phe at 150 rpm and 24 °C was able to produce 1.33 g/L of 2-PE [11].

### 6.8. Other Agro-Industrial Waste

In a study done by Martínez-Avila et al., nine different agro-industrial waste materials were tested as potential substrates for 2-PE production. They included rice husk, brewer’s spent grain, soy fiber, rice fiber, asparagus tails, orange peels, banana peels, green apple pomace, and red apple pomace. SSF technology was used and the experiment was carried out using *P. kudriavzevii* CECT 13184 strain in 0.5 L Erlenmeyer flasks at 30 °C and for up to 96 h fermentation time. When l-Phe was added to the solid substrates, the concentration of 2-PE produced by *P. kudriavzevii* increased by almost 10-fold. However, the addition of sugar beet molasses to the waste materials with significant levels of reducing sugars was not efficient at increasing the 2-PE production in these substrates. 

The highest concentrations of 2-PE produced through the fermentation of these waste materials were: 5.7 mg_2-PE_ per g of dry substrate (g_DS_) after 13 h using rice husk, 6.5 mg_2-PE_ g^−1^_DS_ after 10 h using brewer’s spent grain, 11.5 mg_2-PE_ g^−1^_DS_ after 21 h using soy fiber, 8.5 mg_2-PE_ g^−1^_DS_ after 19 h using rice fiber, 7.7 mg_2-PE_ g^−1^_DS_ after 38 h using asparagus tails, 17.2 mg_2-PE_ g^−1^_DS_ after 78 h using orange peel, 17.2 mg_2-PE_ g^−1^_DS_ after 80 h using banana peel, 17.4 mg_2-PE_ g^−1^_DS_ after 90 h using green apple pomace, and finally 25.2 mg_2-PE_ g^−1^_DS_ after 70 h using red apple pomace. Subsequently, the highest 2-PE levels were produced through the biotransformation of l-Phe using red apple pomace [57].

To summarize, whey, grape must, corn stover, sugar beet molasses, sugarcane bagasse, tobacco, cassava water, red apple pomace, etc., are cheap and abundant agro-industrial by-products that demonstrated to be excellent natural substrates for the production of 2-PE from l-Phe by various yeasts. A comparison of the ability of different yeast species to produce 2-PE via fermentation on synthetic media versus agro-industrial waste and by-products is presented in Table 2. 

## 7. Cytotoxicity of 2-PE

The bioproduction of 2-PE via yeasts is limited by the inhibitory effect of this alcohol on the yeast cells, preventing the accumulation of higher 2-PE contents [28]. In fact, concentrations between 2 and 4 g/L of 2-PE have been found to inhibit the growth of many yeasts species [27]. 2-PE is not equally toxic to all yeasts species and is strain specific. For instance, *K. marxianus* CBS 600 was completely inhibited at 2.6 g/L of 2-PE whereas it took 4 g/L of 2-PE in *S. cerevisiae* GIV 2009 to cause the same effect [39]. It is important to note that in the presence of 2.5 g/L of 2-PE, the growth rate of an Australian ethanol-tolerant *S. cerevisiae* strain, used for cider production, decreased by 75% [77].

Once the reaction system reaches a given concentration of 2-PE, the yeast growth and viability are reduced. Many mechanisms are involved in the inhibition phenomena. First, alcohols increase the cell membrane permeability, promoting the leakage of ions and reduction in the transport of amino acids and glucose [39]. Second, high 2-PE concentration induces a decrease in the respiratory capacity of the cell. Part of this deficiency is attributed to the induction of “petite” mutations due to which yeast cells manufacture non-functional mitochondria, and the other part is caused by a direct inhibition of respiration [77]. In addition, yeasts are not only sensitive to exogenous alcohols but also to the ones produced within the cells, including ethanol. The presence of ethanol, which is a by-product of the yeast fermentation, produces a synergistic effect with 2-PE, amplifying its toxicity [22].

## 8. Conventional Strategies to Increase the 2-PE Production

As previously mentioned, high concentrations of 2-PE inhibit the yeast cell growth. In a process that targets high yield, this inhibition is highly undesirable. There are several ways to overcome the inhibition restraint [40]. Strain mutagenesis and culture medium composition or the optimization of the fermentation conditions are widely used strategies for the improvement of yield. 

First, medium composition rigorously affects the titer and yield of metabolites during the fermentation process. The nitrogen sources, including l-Phe, carbon sources, vitamins and minerals supplemented to the media affect the fermentation process. In addition, the culture temperature, initial medium pH, shaking speed, and fermentation time are all factors that could affect the amount of 2-PE produced [21]. 

Second, mutating the microorganism to increase its tolerance to the product is another way of overcoming the product inhibition. For instance, by overexpressing *ARO8* and *ARO9*, *S. cerevisiae* strain was able to produce 2.61 g/L of 2-PE which is a 36.8% higher yield compared to the wild type strain [56]. Improving 2-PE yield could be attained also using synthetic biology, which is an emerging discipline aiming to implement engineering principles to biological systems in order to make them more controllable and predictable. Particularly, dedicated genetic toolboxes can be expanded in order to artificially generate non-native metabolites [78]. For instance, different strategies have been developed in order to allow engineered *Y. lipolytica* to grow on various cheap substrates, to generate aromatic compounds including 2-PE, and to facilitate product extraction [79].

Third, separating 2-PE from the fermentation medium directly after its biosynthesis, hence allowing the yeasts to continue to produce this aroma compound, is another strategy to overcome product inhibition. This can be achieved by many in situ product recovery (ISPR) techniques [80]. ISPR have various advantages including the prevention of yeast inhibition due to product toxicity, product stabilization, and the easing of downstream processing. Depending on the different separation tools employed in ISPR, the methods can be divided into two-phase extraction, in situ product adsorption, solvent immobilization, and organophilic pervaporation [21]. Among ISPR techniques, two-phase extraction is a simple, inexpensive, and easy to apply at industrial scale. It involves an aqueous phase where the biotransformation occurs and an organic phase where 2-PE extraction continuously takes place [80]. The solvent in the organic phase should present high selectivity towards 2-PE and should perform efficient 2-PE recovery without neither affecting the biotransformation occurring in the aqueous phase nor the quality of the 2-PE being extracted [81]. A suitable extractant solvent should present low viscosity, and should be non-toxic towards yeasts, non-flammable, inexpensive and chemically and thermally stable. In fact, organic solvents including oleic acid, oleyl alcohol, and polypropylene glycol (PPG) 1200 have been successfully reported as suitable solvents [82]. For example, in two-phase fed-batch cultures using oleic acid as an organic solvent, *S. cerevisiae* was able to increase 2-PE production from 4.5 g/L to 12.6 g/L [33]. Moreover, 2-PE production in *K. marxianus* CBS 600 could be increased from 0.9 g/L to 10.2 g/L in two-phase fed-batch culture, using PPG 1200 as extractant [51]. A second type of ISPR techniques is in situ product adsorption (ISPA). ISPA uses macroporous adsorbent resins or other adsorption media in order to facilitate 2-PE product recovery and to avoid substrate inhibition. Polymers have some advantages over organic solvents such as not affecting the final product quality, particularly its organoleptic property. In addition, polymers are non-biodegradable, non-volatile and inexpensive [21]. For instance, using the non-polar macroporous resin D101 in the biotransformation of *S. cerevisiae* BD improved its ability to produce 2-PE with a final concentration of 6.17 g/L. In another study, the hydrophobic cross-linked polystyrene resin HZ818 was used. Seven percent of HZ818 were able to improve the production of 2-PE by 66.2%. In particular, when 7% HZ818 and 12 g/L l-Phe were added to the biotransformation system, *S. cerevisiae* P-3 was able to produce 6.6 g/L of 2-PE [83]. A joint approach of solvent extraction and ISPA could be used to enhance the productivity of the bioconversion. This method is based on the entrapment of an organic solvent, namely dibutylsebacate, into a polymeric matrix of polyethylene in order to form a stable composite resin. Solvent immobilization presented several advantages. It prevented the toxic effect of the solvent on the culture and allowed for an efficient 2-PE removal from the bioreactor without the need for cell separation before the extraction [27]. In this system, *S. cerevisiae* GIV 2009 produced around 3.8 g/L of 2-PE which is 31% higher than that produced by the fed-batch process without solvent immobilization [84]. Additionally, other strategies have also been employed for ISPR including perfusion and pertraction, extraction with ionic liquids, membranes coupled with solvent extraction, etc. All of them aimed to overcome the cytotoxicity limitation of 2-PE and to improve the bioproduction’s yield [74].

**Figure 2:**
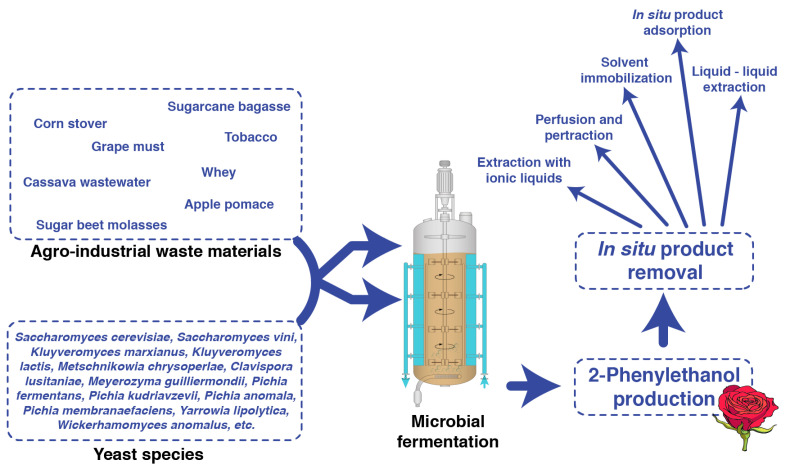
Microbial production of 2-PE. Various agro-industrial waste and by-products serve as substrate for the production of 2-PE through the fermentation of different yeast species.

A summary of the whole process of microbial production of 2-PE is illustrated in Figure 2.

## 9. Conclusions

The aromatic alcohol 2-PE is one of the most used flavors in the food, fragrance, cosmetic and pharmaceutical industries. This rosy-like aroma component has a wide range of applications. The increasing demand for natural products, particularly bio-food and biocosmetics, has led to their universal market growth, hence the need to develop economically profitable production methods. Microbial production of 2-PE, using yeasts appears to be competitive with chemical synthesis and the natural extraction from flowers. Particularly, chemically synthesized 2-PE costs 3.5 US$/kg, naturally synthesized 2-PE (from flowers) costs 1,000 US$/kg, whereas biotechnologically synthesized 2-PE costs around 220 US$/kg. Highly productive yeasts and cheap feedstocks are needed to make the biotechnological route surpass the traditional methods. In fact, since the production of 2-PE is strain specific, various yeast species are able to synthesize 2-PE on synthetic and agro-industrial byproducts, using l-Phe as a substrate, including *S. cerevisiae*, *K. marxianus*, *P. kudriavzevii*, *P. fermentans*, and *Y. lipolytica*. Additionally, due to the abundance, low cost and high nutrient content of agricultural and food waste materials and by-products, they can serve as substrates for 2-PE production. Therefore, the principle of “from waste to product” is applied for obtaining these aroma compounds in a more sustainable and environmentally friendly process. Therefore, reducing not only the price of 2-PE production, but also waste generation. This review presented many agro-industrial by-products that were successfully used as substrates for the biosynthesis of 2-PE, including whey, grape must, corn stover, sugar beet molasses, sugarcane bagasse, tobacco, cassava water, and red apple pomace.

## Figures and Tables

**Table 1:** Advantages and disadvantages of using synthetic media versus agro-industrial waste and by-products for 2-PE production [25,26,54,55].

	Synthetic Culture Media	Agro-Industrial Waste and By-Products
**Advantages**	Many different types are available. Provide all the necessary nutrients for the growth of microorganisms. Composition is known. Give researchers the ability to select and/or differentiate between different species of microorganisms.	Abundant availability. Rich in nutrient composition and bioactive compounds, such as sugars, minerals, and proteins. Offer suitable conditions for the growth of microorganisms, which can use them as nutrients. Can be used as solid support in solid-state fermentation for the production of valuable compounds. Cheap substrate. Hence, their usage as raw materials considerably reduces the production cost. Environmentally friendly, contribute in recycling of waste and in the reduction of solid waste accumulation. Substrates accepted by the European and American legislation for the production of “Natural” products.
**Disadvantages**	Environmentally unfriendly. Expensive due to their constituents. Resource intensive. Are not cost-effective at large-scale, i.e., on an industrial level.	Raw material doesn’t have a constant composition. Due to their complex and unknown components, 2-PE production is still low and unstable. Need usually pretreatments and hydrolysis.

**Table 2:** Comparison of 2-PE production capacity of different yeast species on synthetic media versus agro-industrial waste byproducts.

Synthetic-Based Media				Agro-Industrial Waste and By-Products			
Strain	Media Major Constituents + l-Phe Content	2-PE Production	Ref.	Strain	Media Type + l-Phe Content	2-PE Production	Ref.
***S. cerevisiae***							
JM2014	Medium 8 + 5 g/L l-Phe	3.6 g/L	[43]	JM2014 WUT4 WUT34 WUT35	WGP medium + 0.5% l-Phe	2 g/L	[24]
CWY132	34.16 g/L glucose + 5g/L l-Phe	3.76 g/L	[49]	NS	Tobacco waste + 0 g/L l-Phe	1.55 g/L	[33]
JHY315	20 g/L glucose + 10 g/L l-Phe	4.8 g/L	[51]	NS	Cassava wastewater + 5.5 g/L l-Phe	1.33 g/L	[11]
***K. marxianus***							
CCT 7735	3 g/L glucose + 4 g/L l-Phe	3.44 g/L	[44]	ITD00262	Sweet whey + 0 g/L l-Phe	0.96 g/L	[23]
					Acid whey + 0 g/L l-Phe	0.7 g/L	
					Curd whey + 0 g/L l-Phe	0.47 g/L	
BY25569	20 g/L glucose + 0 g/L l-Phe	1.3 g/L	[52]	CBS 6556	Grape must + 3 g/L l-Phe	0.77 g/L	[4]
				CBS 600	Sugar beet molasses + 7 g/L l-Phe	0.89 g/L	[39]
***Pichia kudriavzevii***							
YF1702	50 g/L glucose + 10.7 g/L l-Phe	5.09 g/L	[47]	WUT7	WGP medium + 0.5% l-Phe	Between 1 and 2 g/L	[24]
				CECT 10467	Sugarcane bagasse + 2% l-Phe	27.2 mg_2-PE_ g^−1^_DS_	[58]
				CECT 13184	Rice husk	5.7 mg_2-PE_ g^−1^_DS_	[57]
					Brewer’s spent grain	6.5 mg_2-PE_ g^−1^_DS_	
					Soy fiber	11.5 mg_2-PE_ g^−1^_DS_	
					Rice fiber	8.5 mg_2-PE_ g^−1^_DS_	
					Asparagus tails	7.7 mg_2-PE_ g^−1^_DS_	
					Orange peel	17.2 mg_2-PE_ g^−1^_DS_	
					Banana peel	17.2 mg_2-PE_ g^−1^_DS_	
					Green apple pomace	17.4 mg_2-PE_ g^−1^_DS_	
					Red apple pomace	25.2 mg_2-PE_ g^−1^_DS_	
